# AI-Driven multi-view learning from CCTA for myocardial infarction diagnosis

**DOI:** 10.1007/s10554-025-03523-6

**Published:** 2025-10-09

**Authors:** Jakub Gwizdala, Adil Salihu, Ortal Senouf, David Meier, David Rotzinger, Salah Qanadli, Olivier Muller, Pascal Frossard, Emmanuel Abbe, Dorina Thanou, Stephane Fournier, Denise Auberson

**Affiliations:** 1 Cardiology Department, University Hospital of Lausanne, University of Lausanne, Lausanne, Switzerland; 2 Institute of Mathematics, School of Computer and Communication Sciences, EPFL, Lausanne, Switzerland; 3 Radiology Department, University Hospital of Lausanne, Lausanne, Switzerland; 4 Signal Processing Laboratory, School of Engineering, EPFL, Lausanne, Switzerland

**Keywords:** Artificial intelligence, Machine learning, CCTA, acute coronary syndrome

## Abstract

Non–ST-elevation acute coronary syndrome (NSTE-ACS) remains a diagnostic challenge, as a proportion of patients do not present with obstructive coronary lesions. Coronary computed tomography angiography (CCTA) has emerged as a non-invasive tool for coronary assessment, and integrating artificial intelligence (AI) may enhance its diagnostic accuracy. This study evaluates a machine learning (ML) model using a learned fusion approach to identify culprit lesions in high-risk NSTE-ACS patients. This study is a sub-analysis of a prospective, multicenter trial including patients with high-risk NSTE-ACS who underwent CCTA, followed by ICA and fractional flow reserve (FFR) assessment in every intermediate stenosis. An ML framework was developed to analyze 2 orthogonal CCTA views of each coronary segment and classify them as culprit or non-culprit, with ICA +/- FFR as gold standards. The model was trained using 5-fold cross-validation and compared against 5 baseline methods, including conventional feature extraction and FFR-CT. Among 80 patients, 514 coronary segments were analyzed, with 63 (12.3%) labeled as culprit. The learned fusion model achieved a sensitivity of 0.55 ± 0.14, specificity of 0.93 ± 0.05, and F1-score of 0.53 ± 0.11. The AUC was 0.84 ± 0.06, matching the performance of FFR-CT (AUC of 0.82 ± 0.08). Our findings demonstrate that the learned fusion approach, based on combining two orthogonal views, achieved a performance level comparable to that of FFR-CT, as shown by the AUC of both techniques. These results confirm that AI-driven CCTA analysis could enhance clinical decision-making in high-risk NSTE-ACS patients, warranting further validation of this method in larger cohorts.

**Graphical abstract:**

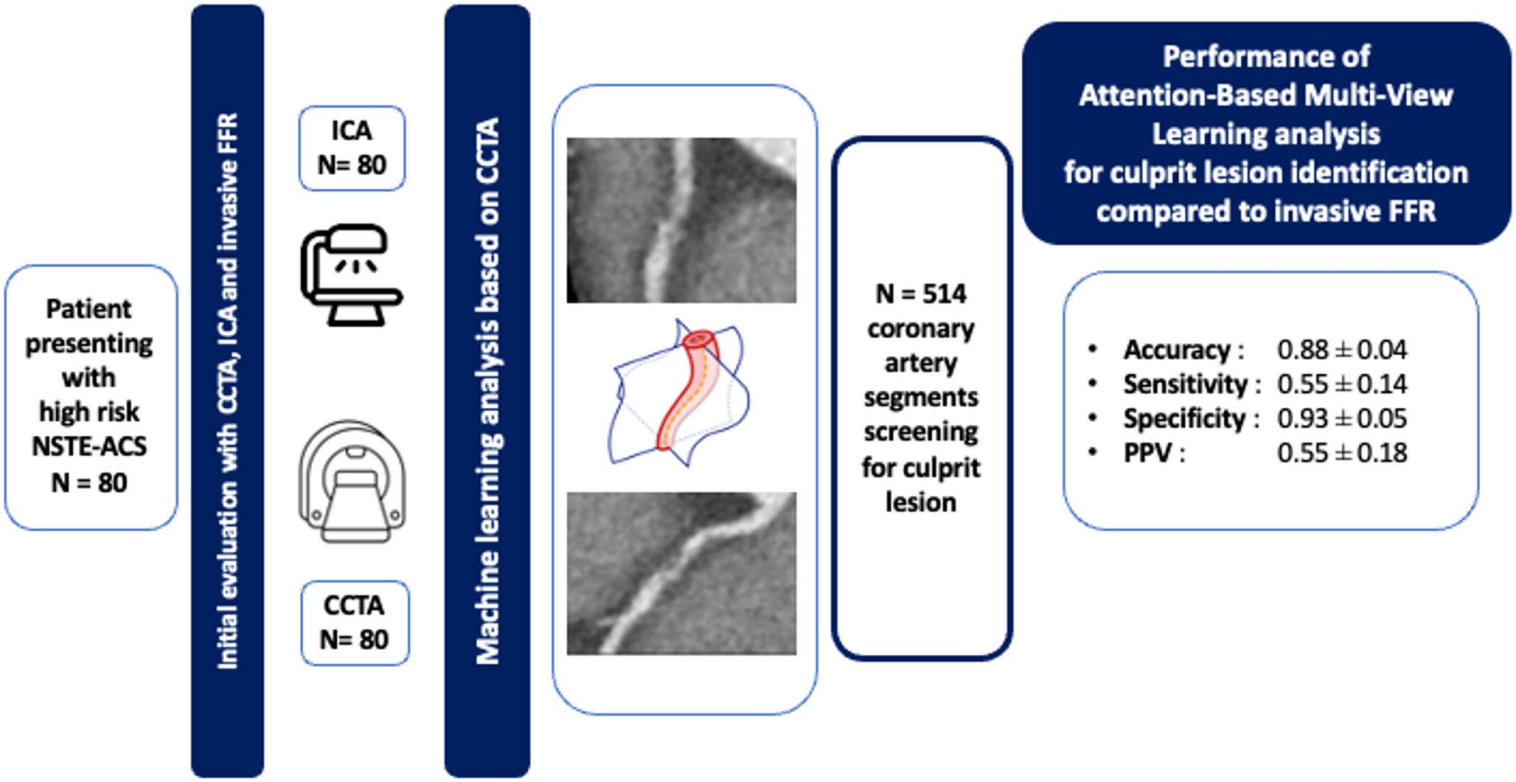

CCTA: coronary computed tomography angiography, FFR: fractional flow reserve, ICA: invasive coronary angiogram, NSTE-ACS: non-ST-elevation acute coronary syndrome PPV: positive predictive value

## Introduction

Acute coronary syndrome (ACS) is one of the most common working diagnoses in cardiology, with coronary angiography being performed in the vast majority of cases. However, the management of non–ST-elevation acute coronary syndrome (NSTE-ACS) remains particularly challenging. According to certain registries, up to 30% of patients presenting with ACS may not have significant coronary lesions [1].

The use of coronary computed tomography angiography (CCTA) has already been incorporated into clinical guidelines for the management of low-risk ACS and chronic coronary syndrome [2–4]. The advent of CCTA has positioned it as a valuable non-invasive alternative for the three-dimensional anatomical assessment of coronary arteries, including plaque composition, morphology, and stenosis quantification [5]. As a complementary tool, fractional flow reserve derived from CCTA (FFR-CT) enables the non-invasive evaluation of the hemodynamic significance of a stenosis [6, 7]. These two approaches (CCTA and FFR-CT) have also been recently studied in high-risk NSTE-ACS, where their performance has been shown to be compatible with potential future clinical implementation [7].

The application of artificial intelligence (AI) is expected to enhance the diagnostic accuracy of various cardiology modalities, including as CCTA, ultimately improving patient management [8, 9]. The use of machine learning (ML) models has demonstrated superior predictive capabilities for ischemic events based on coronary angiography compared to human assessment or angiographic parameters [10–13].

In this study, we aim to assess an ML model based on CCTA findings in the context of high risk NSTE-ACS to accurately identify the presence of a culprit lesion at ICA, thereby improving diagnostic precision and guiding clinical management.

## Methods 

### Clinical study

This study is a sub-analysis of a larger prospective, double-blinded, multicenter trial involving patients presenting to the emergency department with high-risk NSTE-ACS. The study was initially approved in May 2019 by the Medical Ethical Committee of the Canton of Vaud (CER-VD) and is registered on ClinicalTrials.gov (NCT04052763). As part of this study, all patients underwent CCTA, followed by ICA within the same admission, with invasive FFR assessment whenever feasible, in accordance with the latest guidelines [2]. The inclusion and exclusion criteria, along with additional details, can be found in the study description [7]. All patients from our center who underwent a CCTA were included in our sub-study.

### CCTA evaluation

CCTA was performed on a 256-row multidetector CT system (Revolution CT, GE Healthcare). From each CCTA scan, based on a coronary artery centerline extraction, curved multiplanar reconstruction (MPR) views were reconstructed for segments of the three major artery branches: right coronary artery (RCA), segments 1, 2 and 3; left anterior descending coronary artery (LAD), segments 1, 2 and 3; and the first segment of left circumflex artery (LCX). Each segment was visualized with two curved MPR views. The primary view was selected automatically by the hospital software (GE AW Advanced Visualisation) used to generate the reconstructed views. The secondary view was reconstructed in the orthogonal plane to the primary view, adjusted by the medical expert by up to 20°.

Each artery segment was separately labeled as “culprit”, i.e., containing a culprit lesion, or “non-culprit”, i.e., representing a healthy segment or, in some cases, containing a lesion, but one not identified as responsible for the myocardial infarction. This classification of segments and culprit lesion was performed by cardiologist based on the visual assessment of ICA and FFR measurement, when available.

### Deep learning assessment

A machine learning framework based on deep learning was developed to process the two orthogonal views of each arterial segment obtained from CCTA and determine whether a given segment is culprit or not. This framework addresses two main challenges: (i) weak labeling, where labels (i.e., culprit, non-culprit) are assigned at the image level while only specific regions (i.e., lesions) within the image are responsible for the label; and (ii) the representation of the 3D arterial segment using two axial views, ensuring an effective integration of spatial information.

#### Weak labeling treatment

Based on similar studies, we treated this problem by adopting the methods designed for the multiple instance learning framework, where an image is represented by forming a bag of instances associated with one label [14, 15].An interpretation of this framework is particularly suitable for our setup where two views with a single label are available for each concerned artery segment, but the precise location of possible lesions (i.e., instances) inside each view is not provided. In our work the instances are not represented by patches explicitly sampled from the curved MPR views, but by learned features that aggregate information from different regions of the image. This reduces the problem to learning the importance of each region in the image, through a feature attention layer, that is able to highlight information from areas in the image that are relevant for the classification of segments as culprit or not, through an attention score. Such attention mechanisms have been earlier employed in classification tasks in the medical domain, e.g. in Attention Gates [16].

#### Learned two-views fusion

**Fig. 1:**
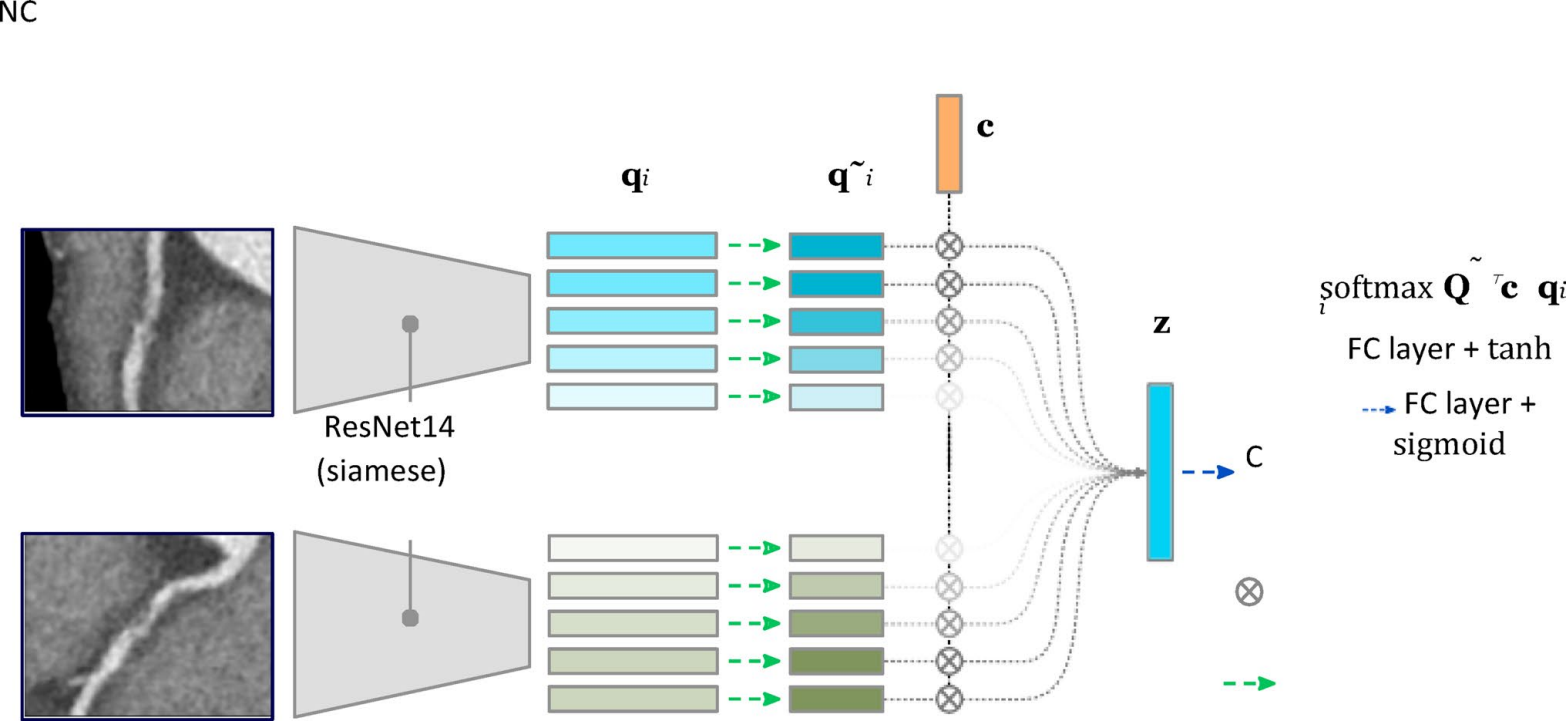
Architecture of the culprit lesion classification model. Both views x^(1)^and x^(2)^ are passed through two Siamese ResNet14 networks to generate feature maps – sets of feature vectors q_i_ arranged spatially over a map with spatial resolution lower than the input image. After projecting to a lower dimensional space and stacking into a Q^˜^matrix, they are combined through the content attention module to generate an artery segment representation. Finally, the classification of the sample as culprit (C) or non-culprit (NC) is performed

Extracting rich information from 3D segments requires considering the orthogonal views of our dataset. We designed a machine learning model that processes both views simultaneously, through a Siamese network. Specifically, in order to extract predictive features from each view, we pass them separately through a Siamese ResNet backbone to obtain two feature maps as shown in Fig. 1 [17]. After computing the feature maps for each view, we fuse them by learning an attention mechanism inspired by the work of Ilse et al. [14]. This method selectively pulls features of higher importance in the feature maps, aggregates them and generates a learned weighted average representation for the artery segment that can be ultimately classified as culprit or non-culprit by a fully-connected layer. The main components of our machine learning model, i.e., the features extraction backbone, together with the views fusion attention layer and the final classifier, were trained using as input the curved MPR views from the CCTA images from the cohort and their corresponding labels in an end-to-end fashion. The objective function used to learn the model parameters is the Focal Loss [18], which mitigates class imbalance by down-weighting well-classified examples and focusing learning on harder, misclassified ones. To further address imbalance, we adopted balanced batch sampling [19], ensuring each mini-batch contains an equal number of samples from the culprit and non-culprit classes.

Due to the scarcity of the available dataset, the data is split into five folds. We use cross-validation to train and validate the performance of the proposed pipeline, a common procedure in machine learning, as shown in Fig. 1. Samples were randomly split into folds patient-wise in a stratified manner, i.e., the ratio between patients with and without culprit segments was similar in each fold. Additionally, we repeated the complete five-fold cross-validation five times, randomizing the order in which the training samples were provided to the model, to further account for the results variability associated with the small dataset size.

This approach was compared to 5 others methods:

(1) *Naïve*, a label is assigned randomly based on the frequency of culprit and non-culprit images in the train set;

(2) *Views as channels*, the two views are stacked in the input’s channels dimension and processed jointly by the same neural network;

(3) *Anomaly detection*, application of the method by Pang et al. [17] where views are still stacked as channels.

(4) *Feature concatenation*, a classic machine learning method for combining views, employing the method by Zhang et al., which concatenates max-pooled feature representations of the two views [20].

(5) *FFR*_*CT*_, FFR values estimated from the CCTA by an external laboratory (HeartFlow ^®^, Redwood City, CA 94063, USA).

## Statistical analysis 

Data were summarized using descriptive statistics. For continuous variables with a normal distribution, the mean and standard deviation (mean ± SD) were reported. For continuous variables that were not normally distributed, the median and interquartile range (IQR) were used. Categorical variables were presented as frequencies and percentages. This study was not designed to perform advanced between-group comparisons. All analyses were performed using SPSS Statistics (IBM).

## Results 

### Clinical data

A total of 86 patients were initially identified in our center. Of these, 80 patients were included in the final analysis. Six patients were excluded due to the inability to transfer imaging data for analysis or insufficient image quality at the time of evaluation. Baseline characteristics of the population can be found in Table 1. The mean age was 62 ± 13 years old with 21 patients being female. Cardiovascular risk factors were as follows: hypercholesterolemia in 49 patients (60%), hypertension in 41 patients (52%), diabetes mellitus in 11 patients (14%). The resulting dataset included 514 coronary artery segments from our 80 patients of which 63 (12.3%) segments were labeled as culprit.

**Table 1:** Baseline characteristics

	Overall *N* = 80
**Clinical data**	
Age, m ± SD (in years)	62 ± 13
Female	22 (28)
Positive family history n (%)	20 (25)
Hypercholesterolemia n (%)	49 (60)
Hypertension n (%)	41 (52)
Diabetes Mellitus n (%)	11 (14)
BMI ≥ 30 kg/m^2^ n (%)	13 (16)
Prior myocardial infarction n (%)	1 (1)
Prior stroke n (%)	3 (4)
Peripheral artery disease n (%)	2 (3)

BMI : Body mass index; SD : standard deviation

### Deep learning analysis

**Fig. 2:**
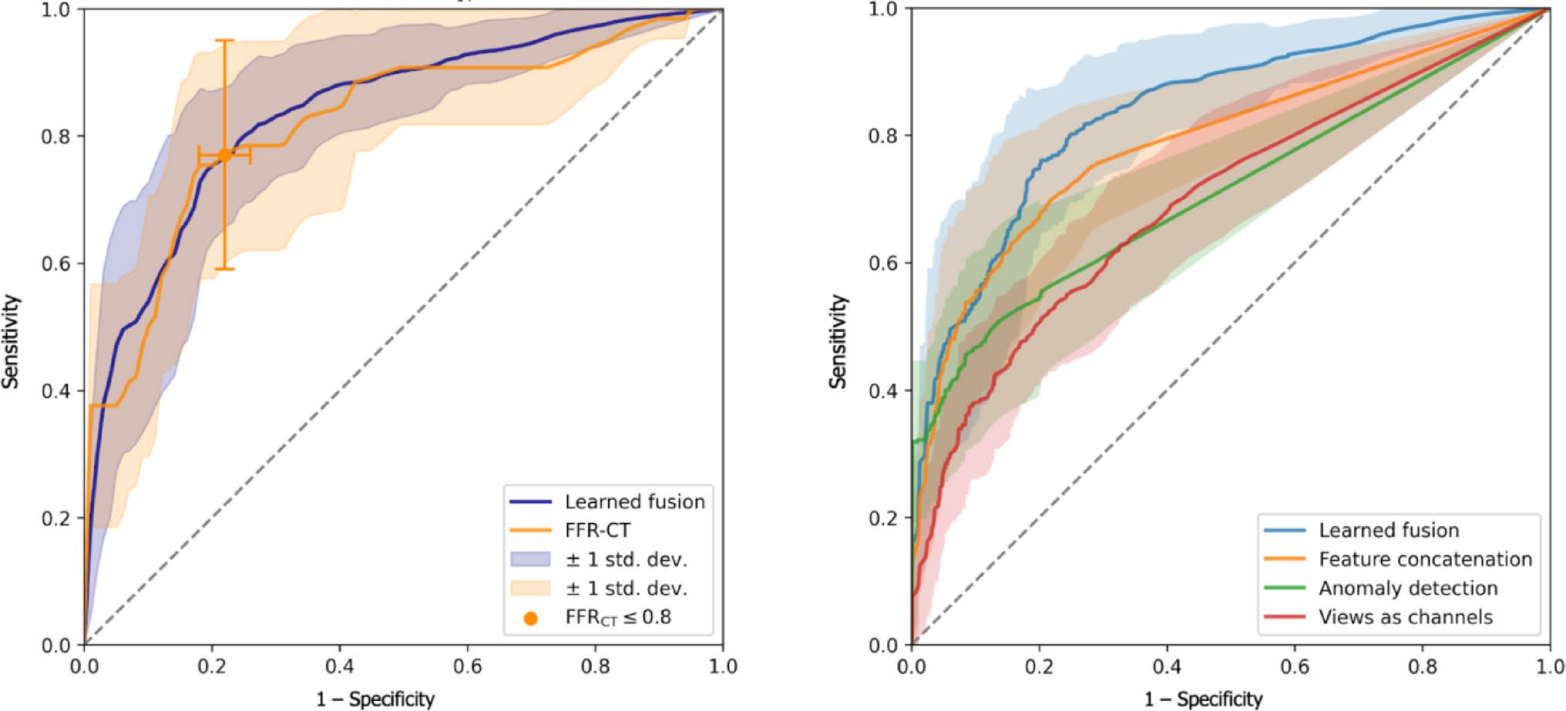
Receiver operating characteristics curve of the different machine learning models and FFR-CT. FFR-CT: fractional flow reserve computed tomography, left graph: ROC curve of the learned fusion model and FFR-CT, right graph: ROC curve of the different machine learning models.

The FFR-CT approach achieved a sensitivity of 0.77 ± 0.18 and a specificity of 0.78 ± 0.04, with an overall accuracy of 0.78 ± 0.04. The F1 score and positive predictive value were 0.46 ± 0.09 and 0.33 ± 0.06, respectively. Our proposed learned fusion model offers a trade-off between sensitivity and specificity depending on the chosen classification threshold. At the threshold set to maximize the F1 score, our model demonstrated a lower sensitivity of 0.55 ± 0.14, but higher specificity of 0.93 ± 0.05 compared to FFR-CT, with an overall accuracy of 0.88 ± 0.04. Its F1 score and positive predictive value were 0.53 ± 0.11 and 0.55 ± 0.18, respectively. Furthermore, by adjusting the threshold, the learned fusion model can achieve a sensitivity of 0.80 ± 0.11 and a specificity of 0.74 ± 0.14. The trade-off between sensitivity and specificity is represented by the ROC curves, where our model’s area under the curve (AUC) was 0.84 ± 0.06 and 0.82 ± 0.08 for FFR-CT. A comparison of our proposed approach with FFR-CT and the other methods of analysis can be found in Table 2; Fig. 2.

**Table 2:** Performance of different machine learning models and FFR-CT

Method	F1	PPV	Sensitivity	Specificity	AUC	Accuracy
Naive	0.12	0.12	0.12	0.88	–	0.78
Views as channels	0.36 ± 0.12	0.39 ± 0.17	0.37 ± 0.14	0.91 ± 0.04	0.70 ± 0.06	0.84 ± 0.03
Anomaly detection	0.40 ± 0.10	0.39 ± 0.10	0.45 ± 0.15	0.90 ± 0.04	0.71 ± 0.08	0.84 ± 0.03
Feature concatenation	0.46 ± 0.13	0.53 ± 0.18	0.46 ± 0.18	**0.93 ± 0.05**	0.79 ± 0.07	0.87 ± 0.04
Learned fusion	**0.53 ± 0.11**	**0.55 ± 0.18**	0.55 ± 0.14	**0.93 ± 0.05**	**0.84 ± 0.06**	**0.88 ± 0.04**
FFRCT	0.46 ± 0.09	0.33 ± 0.06	**0.77 ± 0.18**	0.78 ± 0.04	0.82 ± 0.08	0.78 ± 0.04

AUC: area under the ROC curve, FFR-CT: fractional flow reserve computed tomography, thresholded at 0.8, its AUC was computed under threshold varied in [0, 1] range, PPV: positive predictive value. Bold values indicates the best result for each metric

## Discussion 

This study evaluates a ML-based assessment of CCTA in the particular context of high-risk NSTE-ACS for the identification of culprit lesions. The key findings of this study indicate that, among the methods tested, combining two orthogonal views, through a fusion process that is learned from the data, appears to be the A-based computer vision approach with the most encouraging performance for identifying culprit lesions.

The use of AI in the decision-making process is becoming increasingly important [21]. ML enables automated quantification of stenosis as well as precise assessment of plaque physiology or even for its predictive value for future myocardial infarction [22–25]. AI models have demonstrated strong performance in patients with stable coronary artery disease, achieving an accuracy as high as 86% in identifying coronary stenosis greater than 70% [22]. However, the mere presence of coronary stenosis does not determine whether treatment is necessary or whether the lesion is the culprit of the myocardial infarction. To address this limitation, it is essential to assess either the hemodynamic impact of these lesions, or adopt an ML-based approach to identify patterns indicative of the culprit lesion.

The use of FFR-CT provides not only an anatomical assessment but also an assessment of the hemodynamic impact of the lesion. Our recent study on the use of FFR-CT in the context of NSTE-ACS demonstrated superior accuracy compared to CCTA alone for identifying significant lesions (AUC: 0.84 vs. 0.65, respectively; *p* < 0.01). The learned fusion model that was introduced in this work demonstrates comparable performance to that of FFR-CT, with an AUC of 0.84 ± 0.06 and 0.82 ± 0.08 respectively (Table 2; Fig. 2). At the threshold maximizing the F1 score, the model achieves a high specificity at the expense of sensitivity. This demonstrates its possible use as a confirmatory test, i.e. confirming the presence of culprit lesions in patients with positive findings, thereby reinforcing the decision for invasive angiography. In comparison, our baseline ML approaches have not yielded equally performant models to justify prioritizing their use, as shown in Table 2. Other multi-view fusion for the assessment of stenosis in coronary angiogram images has already been implemented; however, the fixed fusion method demonstrated potential [20].

Coronary artery assessment approaches usually rely on the multiplanar segmentation method which have demonstrated their effectiveness in evaluating coronary stenosis [26–28]. In our study, we use two reconstructed cross-sectional views to represent a coronary segment. Our findings suggest that in this setting, aggregating information from the two views with the learned fusion approach provides encouraging results for the identification of culprit lesions in the context of ACS. The implementation of such a model could facilitate the development of a real-time, local computational tool for analyzing CCTA images, which would assist in the decision-making process to refer for invasive coronary angiography. This approach could be further enhanced by incorporating additional cross-sectional views and clinical data to improve the model’s accuracy.

## Limitations 

The primary limitation of our study is the relatively small sample size, which affects the generalizability and statistical power of our findings. While our learned fusion approach demonstrated potential, external validation of the model in larger, multi-center cohorts will be crucial to ensure their reproducibility and clinical applicability. Additionally, due to technical constraints, we decided not to include segments 2 and 3 of the circumflex coronary artery. This decision was made due to the presence of small-caliber vessels in these segments, which were excluded for consistency with other patients. Finally, the initial visual classification of lesions, although performed by experienced cardiologists, remains inherently subjective and may introduce some degree of interobserver variability.

## Conclusion 

In this study, we evaluated a ML-based approach for identifying culprit lesions in high-risk NSTE-ACS patients using CCTA. Our findings indicate that our method, which is based on learning to combine two orthogonal views of CCTA scans achieves superior accuracy compared to other tested baseline methods. This study highlights the potential that AI has to improve non-invasive coronary artery assessment, paving the way for more precise and individualized patient management. Future research should focus on validating our approach in larger cohorts and exploring the integration of additional clinical and imaging data to further refine its predictive capabilities.

## Data Availability

The data underlying this article will be shared on reasonable request to the corresponding author.
